# Neuroferritinopathy: iron in the brain

**DOI:** 10.1186/1755-8166-7-S1-I36

**Published:** 2014-01-21

**Authors:** John Burn

**Affiliations:** 1 Institute of Genetic Medicine, Newcastle University, International Centre for Life, Newcastle upon Tyne, UK

## 

Careful attention to clinical phenotypes can identify new diseases which are now amenable to molecular genetic elucidation. In the late 1980's I met a family labelled as having Huntington's disease but with absence of dementia. Scanning revealed unusual cavitation of the basal ganglia. The pedigree was extended my connection to a second family using birth records. We mapped the gene to chromosome 19 and went on to identify an unusual mutation in the E helix of light chain ferritin. Staining for iron revealed huge accumulation of iron/ferritin complexes in the brain leading to neurodegeneration. We named the condition neuroferritinopathy and tested desferrioxamine without success. We have shown that accumulation of iron commences in childhood and are now preparing to test deferiprone as an iron chelator which crosses the blood brain barrier.

